# The genome sequence of the John Dory, *Zeus faber *Linnaeus, 1758

**DOI:** 10.12688/wellcomeopenres.21140.1

**Published:** 2024-03-19

**Authors:** Patrick Adkins, Joanna Harley, Rachel Brittain, Kesella Scott-Somme, Freja Azzopardi, Marine Biological Association Genome Acquisition Lab, Darwin Tree of Life Barcoding collective, Wellcome Sanger Institute Tree of Life Management, Samples and Laboratory team, Wellcome Sanger Institute Scientific Operations: Sequencing Operations, Wellcome Sanger Institute Tree of Life Core Informatics team, Tree of Life Core Informatics collective, Darwin Tree of Life Consortium

**Affiliations:** 1 The Marine Biological Association, Plymouth, England, UK

**Keywords:** Zeus faber, John Dory, genome sequence, chromosomal, Zeiformes

## Abstract

We present a genome assembly from an individual *Zeus faber* (the John Dory; Chordata; Actinopteri; Zeiformes; Zeidae). The genome sequence is 804.7 megabases in span. Most of the assembly is scaffolded into 22 chromosomal pseudomolecules. The mitochondrial genome has also been assembled and is 16.72 kilobases in length.

## Species taxonomy

Eukaryota; Opisthokonta; Metazoa; Eumetazoa; Bilateria; Deuterostomia; Chordata; Craniata; Vertebrata; Gnathostomata; Teleostomi; Euteleostomi; Actinopterygii; Actinopteri; Neopterygii; Teleostei; Osteoglossocephalai; Clupeocephala; Euteleosteomorpha; Neoteleostei; Eurypterygia; Ctenosquamata; Acanthomorphata; Paracanthopterygii; Zeiogadaria; Zeariae; Zeiformes; Zeidae; *Zeus*; *Zeus faber* Linnaeus, 1758 (NCBI:txid64108).

## Background

John Dory, *Zeus faber* Linnaeus, 1758, also known as St Peter’s fish, is a solitary, demersal marine fish characterised by a laterally compressed body of golden-brown colouration with a black spot on either side and long dorsal spines ( Wheeler, 1978). It is widely distributed in the eastern Atlantic, Mediterranean, Pacific and Indian Oceans, and along the entire West African coast, typically occupying depths of 0–200 m ( Iwamoto, 2015; Maravelias *et al.*, 2007). Recently, it has been recorded for the first time in the Black Sea ( Aydın & Karadurmuş, 2023). John Dory has a large, highly protrusible mouth allowing it to predate on relatively large fish which they target using well-developed eyes ( Kim *et al.*, 2020; Stergiou & Fourtouni, 1991).

In the eastern Mediterranean, John Dory begins its life feeding on small zooplankton, such as mysids, until it reaches a total length (TL) of approximately 80 mm ( Stergiou & Fourtouni, 1991). It gradually switches to small benthopelagic fishes then, as it grows, it predates on larger schooling pelagic teleosts ( Kim *et al.*, 2020; Stergiou & Fourtouni, 1991). In Korean coastal waters there is also varying diet composition with size and age ( Kim *et al.*, 2020). Off the Portuguese coast however, there is no prey switching from juvenile to adult life stages ( Silva, 1999). Predominantly, evidence suggests it is an opportunistic feeder, switching prey depending on food availability and abundance which can vary seasonally and with life stage ( Kim *et al.*, 2020). Recently, meso- and microplastics have been found to occur in the gastrointestinal tracts of *Z. faber* in the Mediterranean ( Bottari *et al.*, 2019).

*Z. faber* is known to make ‘croaking’ or ‘barking’ noises upon capture onboard ( Radford *et al.*, 2018). These vocalisations have since been documented *in-situ* in Australia and were found to induce an escape response in conspecifics and heterospecifics such as the Australian Snapper ( *Pagurus auratus*), suggesting they make sounds as a territorial display against competitors ( Radford *et al.*, 2018).

John Dory is a commercially important species valued for human consumption, meal, and fish oil, as well as being important as a gamefish and in the aquarium trade ( Iwamoto, 2015). It has been a prominent species in mixed species trawl fisheries in the British Isles and is a notable by-catch species for trawl gears globally ( Dunn, 2001; Iwamoto, 2015). Despite being a highly regarded food fish, the only stock assessment for the British Isles, to our knowledge, was carried out between April 1994 and March 1996 ( Dunn, 2001). Dunn describes landings from commercial fisheries in England and Wales alongside biological samples and catch data from the English Channel. John Dory was most abundant in the south and southwest of the British Isles ( Dunn, 2001; Wheeler, 1969). Evidence suggests that the English Channel is a seasonal nursery ground for *Z. faber*: seasonal peaks in landings coincided with the period of recruitment, in this case during quarters three and four, when individuals were just over 1 year old at the transition between juvenile and adult life stages species ( Dunn, 2001). Recruitment total length of the species was approximately 23 cm TL. Most commercial landings were in the range 23–29 cm TL with a maximum observed TL of 59 cm. Mean TL of first maturity was approximately 26 cm for males and 34.5 cm for females. The global conservation status of this species was last assessed in 2013, deemed ‘Data Deficient’ on the IUCN Red List ( Iwamoto, 2015), which reflects the lack of historical data and biological information for this species ( Dunn, 2001). Because of this, stock status and fishing pressure is uncertain for *Z. faber*.

In the Northeast Atlantic (FAO area 27), consuming John Dory may prove harmful to human health, as mercury levels of 0.68 ± 0.07 μg g ^−1^ exceed the maximum limit deemed safe for human consumption (0.5 μg g ^−1^) ( Vieira *et al.*, 2021). John Dory imported from Senegal and sold in Turkish fish markets, as well as individuals caught around Turkey, have also been found to carry larvae of the nematode *Anisakis pegreffii*, with potential implications for human health if consumed ( Pekmezci, 2019; Yardimci *et al.*, 2014).

Molecular investigation of this species has shown significant genetic differentiation (7.44%) between clades in the North Atlantic/Mediterranean region and Australasia, indicating the possibility that they have speciated ( Ward *et al.*, 2008). The first genome of *Z. faber* was generated in 2016 for a study that suggests immune-related genes play an important role in teleost evolution and speciation ( Malmstrøm *et al.*, 2016). Here we present the second published genome of John Dory, collected and sequenced as part of the Darwin Tree of Life project ( Blaxter *et al.*, 2022). This dataset will be important for furthering our understanding of teleost pathology, immunology, evolution and phylogenetics ( Malmstrøm *et al.*, 2016; Ward *et al.*, 2008).

## Genome sequence report

**Figure 1.**
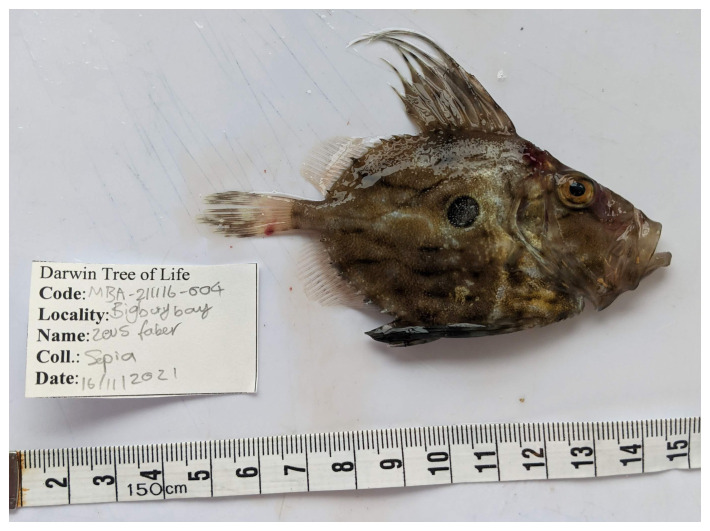
Photograph of the *Zeus faber* (fZeuFab8) specimen used for genome sequencing.

The genome was sequenced from an individual *Zeus faber* ( Figure 1) collected from Bigbury Bay, UK (50.27, –3.97). A total of 43-fold coverage in Pacific Biosciences single-molecule HiFi long reads was generated. Primary assembly contigs were scaffolded with chromosome conformation Hi-C data. Manual assembly curation corrected 9 missing joins or mis-joins, reducing the scaffold number by 1.04%.

**Figure 2.**
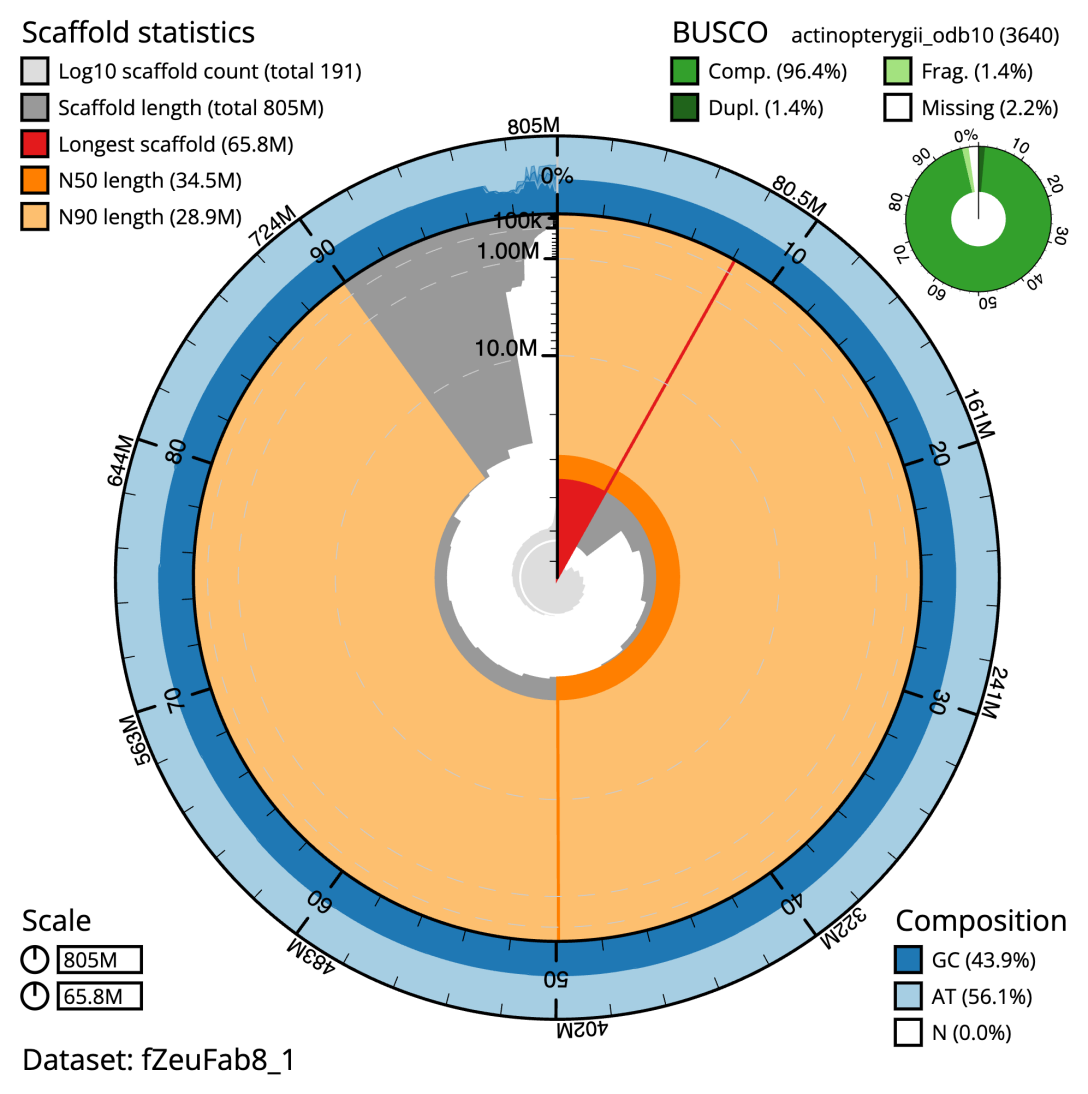
Genome assembly of *Zeus faber*, fZeuFab8.1: metrics. The BlobToolKit snail plot shows N50 metrics and BUSCO gene completeness. The main plot is divided into 1,000 size-ordered bins around the circumference with each bin representing 0.1% of the 804,731,948 bp assembly. The distribution of scaffold lengths is shown in dark grey with the plot radius scaled to the longest scaffold present in the assembly (65,762,550 bp, shown in red). Orange and pale-orange arcs show the N50 and N90 scaffold lengths (34,476,449 and 28,869,016 bp), respectively. The pale grey spiral shows the cumulative scaffold count on a log scale with white scale lines showing successive orders of magnitude. The blue and pale-blue area around the outside of the plot shows the distribution of GC, AT and N percentages in the same bins as the inner plot. A summary of complete, fragmented, duplicated and missing BUSCO genes in the actinopterygii_odb10 set is shown in the top right. An interactive version of this figure is available at https://blobtoolkit.genomehubs.org/view/fZeuFab8_1/dataset/fZeuFab8_1/snail.

**Figure 3.**
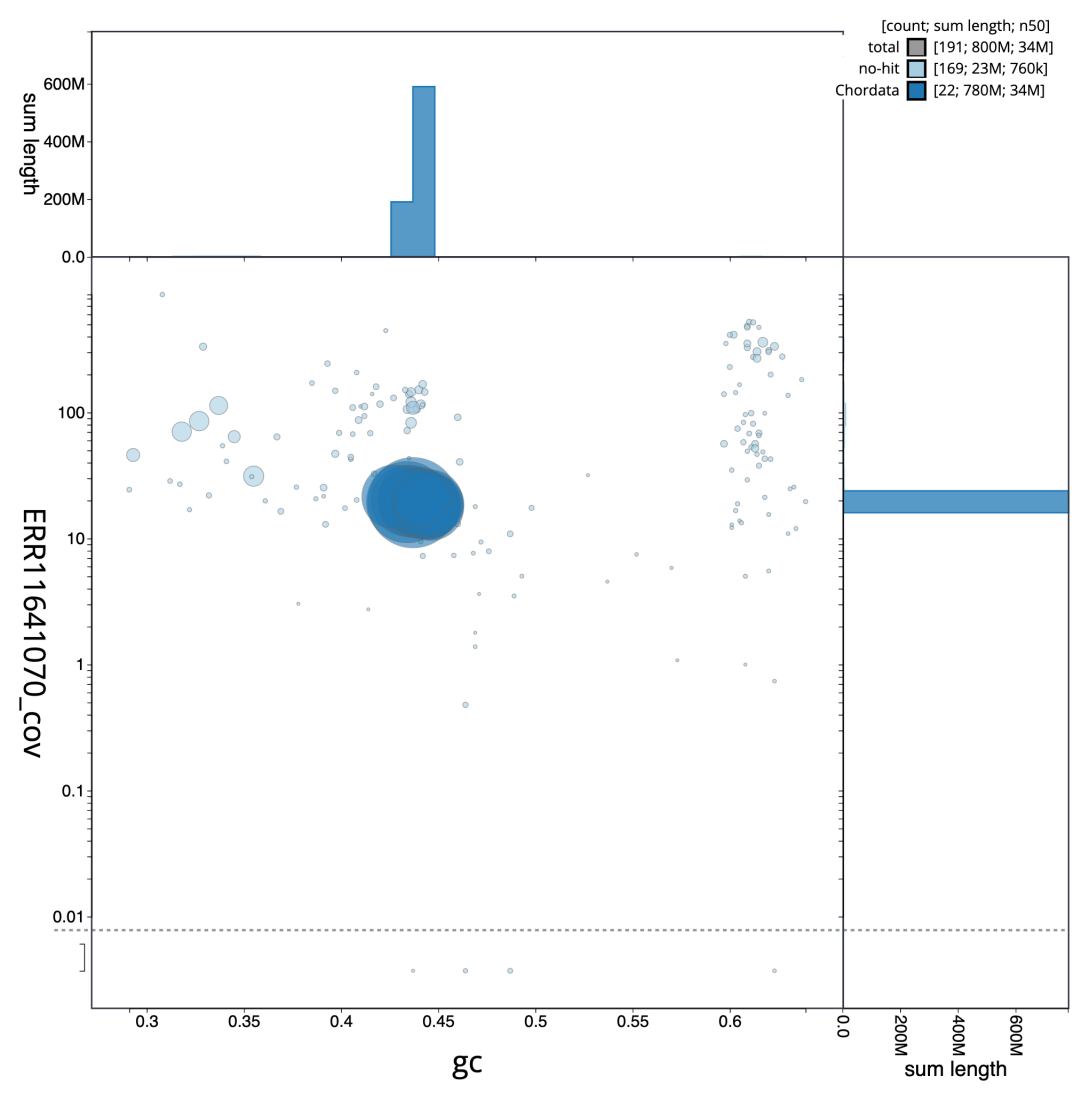
Genome assembly of *Zeus faber*, fZeuFab8.1: BlobToolKit GC-coverage plot. Sequences are coloured by phylum. Circles are sized in proportion to sequence length. Histograms show the distribution of sequence length sum along each axis. An interactive version of this figure is available at https://blobtoolkit.genomehubs.org/view/fZeuFab8_1/dataset/fZeuFab8_1/blob.

**Figure 4.**
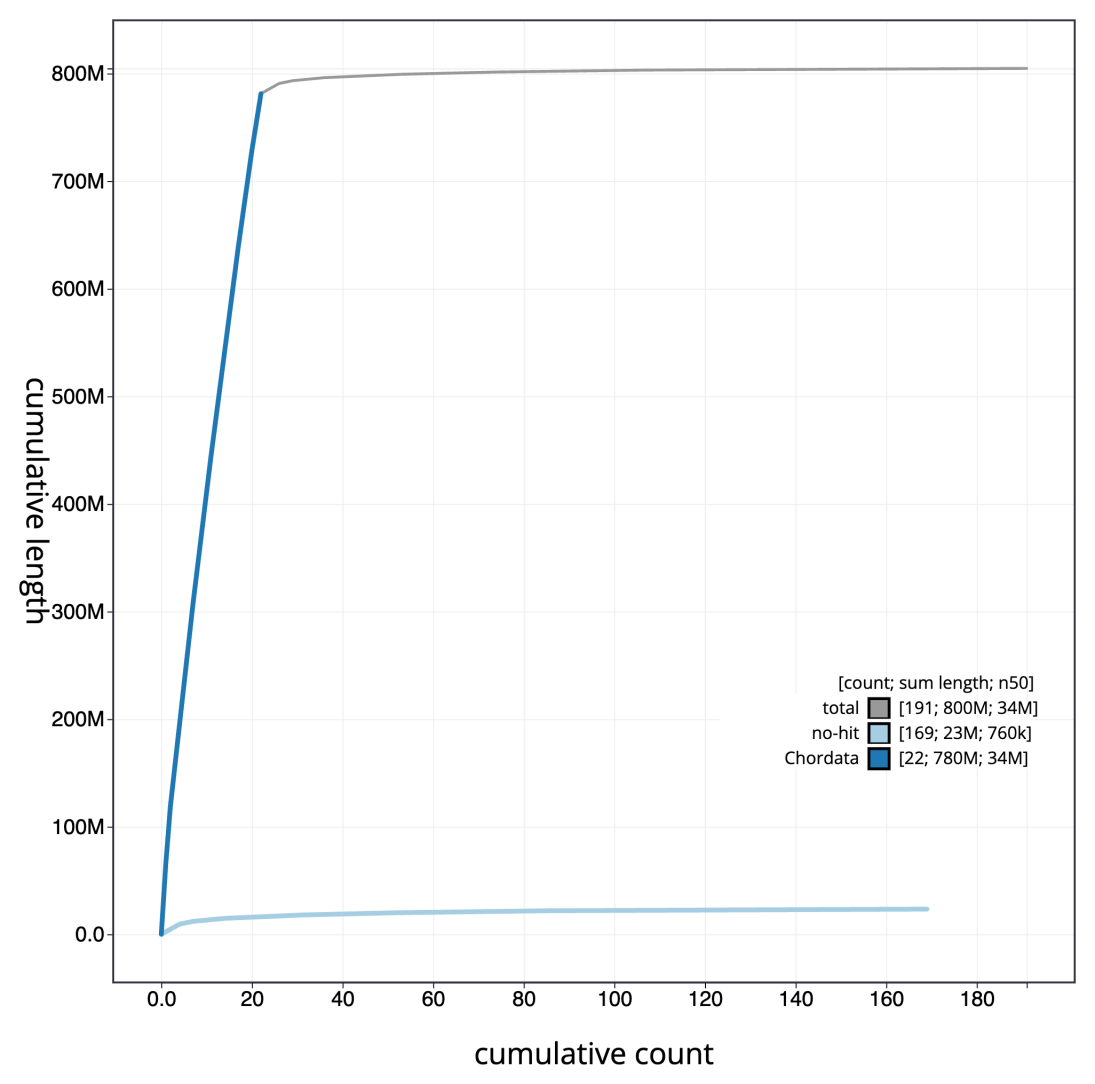
Genome assembly of *Zeus faber*, fZeuFab8.1: BlobToolKit cumulative sequence plot. The grey line shows cumulative length for all sequences. Coloured lines show cumulative lengths of sequences assigned to each phylum using the buscogenes taxrule. An interactive version of this figure is available at https://blobtoolkit.genomehubs.org/view/fZeuFab8_1/dataset/fZeuFab8_1/cumulative.

**Figure 5.**
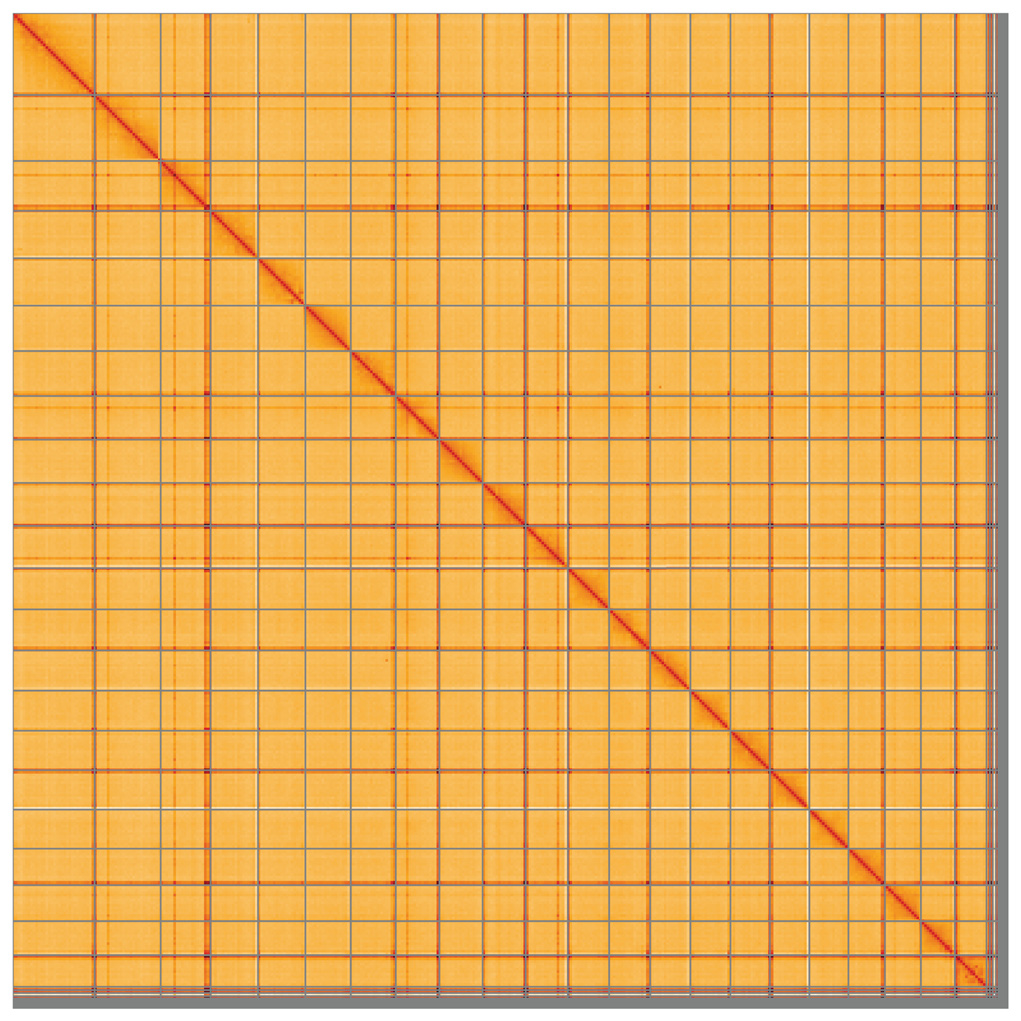
Genome assembly of *Zeus faber*, fZeuFab8.1: Hi-C contact map of the fZeuFab8.1 assembly, visualised using HiGlass. Chromosomes are shown in order of size from left to right and top to bottom. An interactive version of this figure may be viewed at https://genome-note-higlass.tol.sanger.ac.uk/l/?d=ODHdK-fnRfy3tLmo69JGwQ.

The final assembly has a total length of 804.7 Mb in 190 sequence scaffolds with a scaffold N50 of 34.5 Mb ( Table 1). The snail plot in Figure 2 provides a summary of the assembly statistics, while the distribution of assembly scaffolds on GC proportion and coverage is shown in Figure 3. The cumulative assembly plot in Figure 4 shows curves for subsets of scaffolds assigned to different phyla. Most (97.08%) of the assembly sequence was assigned to 22 chromosomal-level scaffolds. Chromosome-scale scaffolds confirmed by the Hi-C data are named in order of size ( Figure 5; Table 2). While not fully phased, the assembly deposited is of one haplotype. Contigs corresponding to the second haplotype have also been deposited. The mitochondrial genome was also assembled and can be found as a contig within the multifasta file of the genome submission.

**Table 1.** Genome data for *Zeus faber*, fZeuFab8.1.

Project accession data		
Assembly identifier	fZeuFab8.1	
Species	*Zeus faber*	
Specimen	fZeuFab8	
NCBI taxonomy ID	64108	
BioProject	PRJEB63619	
BioSample ID	SAMEA111562156	
Isolate information	fZeuFab8 (DNA, Hi-C and RNA sequencing)	
Assembly metrics *		*Benchmark*
Consensus quality (QV)	52.6	*≥ 50*
*k*-mer completeness	99.98%	*≥ 95%*
BUSCO **	C:96.4%[S:94.9%,D:1.4%], F:1.4%,M:2.2%,n:3,640	*C ≥ 95%*
Percentage of assembly mapped to chromosomes	97.08%	*≥ 95%*
Sex chromosomes	None	*localised* *homologous pairs*
Organelles	Mitochondrial genome: 16.72 kb	*complete single* *alleles*
Raw data accessions		
PacificBiosciences SEQUEL II	ERR11641070, ERR11641069	
Hi-C Illumina	ERR11641144, ERR11641145	
PolyA RNA-Seq Illumina	ERR11641143	
Genome assembly		
Assembly accession	GCA_960531495.1	
*Accession of alternate* *haplotype*	GCA_960530785.1	
Span (Mb)	804.7	
Number of contigs	1,078	
Contig N50 length (Mb)	1.4	
Number of scaffolds	190	
Scaffold N50 length (Mb)	34.5	
Longest scaffold (Mb)	65.76	

* Assembly metric benchmarks are adapted from column VGP-2020 of “Table 1: Proposed standards and metrics for defining genome assembly quality” from Rhie *et al.* (2021). ** BUSCO scores based on the actinopterygii_odb10 BUSCO set using version 5.3.2. C = complete [S = single copy, D = duplicated], F = fragmented, M = missing, n = number of orthologues in comparison. A full set of BUSCO scores is available at https://blobtoolkit.genomehubs.org/view/fZeuFab8_1/dataset/fZeuFab8_1/busco.

**Table 2.** Chromosomal pseudomolecules in the genome assembly of *Zeus faber*, fZeuFab8.

INSDC accession	Chromosome	Length (Mb)	GC%
OY482845.1	1	65.76	43.5
OY482846.1	2	52.95	43.5
OY482847.1	3	40.01	43.5
OY482848.1	4	38.03	43.5
OY482849.1	5	37.98	44.0
OY482850.1	6	36.47	44.5
OY482851.1	7	36.1	44.0
OY482852.1	8	35.01	44.0
OY482853.1	9	34.68	44.5
OY482854.1	10	34.48	44.0
OY482855.1	11	33.96	43.5
OY482856.1	12	33.1	44.0
OY482857.1	13	32.89	44.5
OY482858.1	14	32.3	43.5
OY482859.1	15	32.12	44.5
OY482860.1	16	31.67	44.5
OY482861.1	17	31.65	42.5
OY482862.1	18	31.31	44.5
OY482863.1	19	29.26	44.0
OY482864.1	20	28.87	44.5
OY482865.1	21	27.29	45.0
OY482866.1	22	25.41	44.0
OY482867.1	MT	0.02	42.5

The estimated Quality Value (QV) of the final assembly is 52.6 with *k*-mer completeness of 99.98%, and the assembly has a BUSCO v5.3.2 completeness of 96.4% (single = 94.9%, duplicated = 1.4%), using the actinopterygii_odb10 reference set ( *n* = 3,640).

Metadata for specimens, barcode results, spectra estimates, sequencing runs, contaminants and pre-curation assembly statistics are given at https://links.tol.sanger.ac.uk/species/64108.

## Methods

### Sample acquisition and nucleic acid extraction

A *Zeus faber* specimen (specimen ID MBA-211116-004A, ToLID fZeuFab8) was collected from Bigbury Bay, UK (latitude 50.27, longitude –3.97) on 2021-11-16 using an otter trawl deployed from the RV Sepia. The collectors were Patrick Adkins, Joanna Harley, Rachel Brittain, Kesella Scott-Somme (all Marine Biological Association) and identified by Rachel Brittain, and then preserved in liquid nitrogen. The fish died as part of a trawl attached to another project and was opportunistically taken and dissected by the DToL team who were also on board the Sepia that day.

The workflow for high molecular weight (HMW) DNA extraction at the Wellcome Sanger Institute (WSI) includes a sequence of core procedures: sample preparation; sample homogenisation, DNA extraction, fragmentation, and clean-up. In sample preparation, the fZeuFab8 sample was weighed and dissected on dry ice ( Jay *et al.*, 2023). Tissue was homogenised using a PowerMasher II tissue disruptor ( Denton *et al.*, 2023a). HMW DNA was extracted in the WSI Scientific Operations core using the Automated MagAttract v2 protocol ( Oatley *et al.*, 2023). The DNA was sheared into an average fragment size of 12–20 kb in a Megaruptor 3 system with speed setting 31 ( Bates *et al.*, 2023). Sheared DNA was purified by solid-phase reversible immobilisation ( Strickland *et al.*, 2023): in brief, the method employs a 1.8X ratio of AMPure PB beads to sample to eliminate shorter fragments and concentrate the DNA. The concentration of the sheared and purified DNA was assessed using a Nanodrop spectrophotometer and Qubit Fluorometer and Qubit dsDNA High Sensitivity Assay kit. Fragment size distribution was evaluated by running the sample on the FemtoPulse system.

RNA was extracted from tissue of fZeuFab8 in the Tree of Life Laboratory at the WSI using the RNA Extraction: Automated MagMax™ *mir*Vana protocol ( do Amaral *et al.*, 2023). The RNA concentration was assessed using a Nanodrop spectrophotometer and a Qubit Fluorometer using the Qubit RNA Broad-Range Assay kit. Analysis of the integrity of the RNA was done using the Agilent RNA 6000 Pico Kit and Eukaryotic Total RNA assay.

Protocols developed by the WSI Tree of Life laboratory are publicly available on protocols.io ( Denton *et al.*, 2023b).

### Sequencing

Pacific Biosciences HiFi circular consensus DNA sequencing libraries were constructed according to the manufacturers’ instructions. Poly(A) RNA-Seq libraries were constructed using the NEB Ultra II RNA Library Prep kit. DNA and RNA sequencing was performed by the Scientific Operations core at the WSI on Pacific Biosciences SEQUEL II (HiFi) and Illumina NovaSeq 6000 (RNA-Seq) instruments. Hi-C data were also generated from tissue of fZeuFab8 using the Arima2 kit and sequenced on the Illumina NovaSeq 6000, Illumina NovaSeq 6000 instrument.

### Genome assembly, curation and evaluation

Assembly was carried out with Hifiasm ( Cheng *et al.*, 2021) and haplotypic duplication was identified and removed with purge_dups ( Guan *et al.*, 2020). The assembly was then scaffolded with Hi-C data ( Rao *et al.*, 2014) using YaHS ( Zhou *et al.*, 2023). The assembly was checked for contamination and corrected as described previously ( Howe *et al.*, 2021). Manual curation was performed using HiGlass ( Kerpedjiev *et al.*, 2018) and PretextView ( Harry, 2022). The mitochondrial genome was assembled using MitoHiFi ( Uliano-Silva *et al.*, 2023), which runs MitoFinder ( Allio *et al.*, 2020) or MITOS ( Bernt *et al.*, 2013) and uses these annotations to select the final mitochondrial contig and to ensure the general quality of the sequence.

A Hi-C map for the final assembly was produced using bwa-mem2 ( Vasimuddin *et al.*, 2019) in the Cooler file format ( Abdennur & Mirny, 2020). To assess the assembly metrics, the *k*-mer completeness and QV consensus quality values were calculated in Merqury ( Rhie *et al.*, 2020). This work was done using Nextflow ( Di Tommaso *et al.*, 2017) DSL2 pipelines “sanger-tol/readmapping” ( Surana *et al.*, 2023a) and “sanger-tol/genomenote” ( Surana *et al.*, 2023b). The genome was analysed within the BlobToolKit environment ( Challis *et al.*, 2020) and BUSCO scores ( Manni *et al.*, 2021; Simão *et al.*, 2015) were calculated.

Table 3 contains a list of relevant software tool versions and sources.

**Table 3.** Software tools: versions and sources.

Software tool	Version	Source
BlobToolKit	4.1.7	https://github.com/blobtoolkit/blobtoolkit
BUSCO	5.3.2	https://gitlab.com/ezlab/busco
Hifiasm	0.19.5-r587	https://github.com/chhylp123/hifiasm
HiGlass	1.11.6	https://github.com/higlass/higlass
Merqury	MerquryFK	https://github.com/thegenemyers/MERQURY.FK
MitoHiFi	3	https://github.com/marcelauliano/MitoHiFi
PretextView	0.2	https://github.com/wtsi-hpag/PretextView
purge_dups	1.2.5	https://github.com/dfguan/purge_dups
sanger-tol/ genomenote	v1.0	https://github.com/sanger-tol/genomenote
sanger-tol/ readmapping	1.1.0	https://github.com/sanger-tol/readmapping/tree/1.1.0
YaHS	1.2a.2	https://github.com/c-zhou/yahs

### Wellcome Sanger Institute – Legal and Governance

The materials that have contributed to this genome note have been supplied by a Darwin Tree of Life Partner. The submission of materials by a Darwin Tree of Life Partner is subject to the **‘Darwin Tree of Life Project Sampling Code of Practice’**, which can be found in full on the Darwin Tree of Life website here. By agreeing with and signing up to the Sampling Code of Practice, the Darwin Tree of Life Partner agrees they will meet the legal and ethical requirements and standards set out within this document in respect of all samples acquired for, and supplied to, the Darwin Tree of Life Project.

Further, the Wellcome Sanger Institute employs a process whereby due diligence is carried out proportionate to the nature of the materials themselves, and the circumstances under which they have been/are to be collected and provided for use. The purpose of this is to address and mitigate any potential legal and/or ethical implications of receipt and use of the materials as part of the research project, and to ensure that in doing so we align with best practice wherever possible. The overarching areas of consideration are:

•      Ethical review of provenance and sourcing of the material

•      Legality of collection, transfer and use (national and international)

Each transfer of samples is further undertaken according to a Research Collaboration Agreement or Material Transfer Agreement entered into by the Darwin Tree of Life Partner, Genome Research Limited (operating as the Wellcome Sanger Institute), and in some circumstances other Darwin Tree of Life collaborators.

## Data Availability

European Nucleotide Archive: *Zeus faber* (John dory). Accession number PRJEB63619; https://identifiers.org/ena.embl/PRJEB63619 ( Wellcome Sanger Institute, 2023). The genome sequence is released openly for reuse. The *Zeus faber* genome sequencing initiative is part of the Darwin Tree of Life (DToL) project. All raw sequence data and the assembly have been deposited in INSDC databases. The genome will be annotated using available RNA-Seq data and presented through the Ensembl pipeline at the European Bioinformatics Institute. Raw data and assembly accession identifiers are reported in Table 1.

## Author information

Members of the Marine Biological Association Genome Acquisition Lab are listed here: https://doi.org/10.5281/zenodo.8382513.

Members of the Darwin Tree of Life Barcoding collective are listed here: https://doi.org/10.5281/zenodo.4893703.

Members of the Wellcome Sanger Institute Tree of Life Management, Samples and Laboratory team are listed here: https://doi.org/10.5281/zenodo.10066175.

Members of Wellcome Sanger Institute Scientific Operations: Sequencing Operations are listed here: https://doi.org/10.5281/zenodo.10043364.

Members of the Wellcome Sanger Institute Tree of Life Core Informatics team are listed here: https://doi.org/10.5281/zenodo.10066637.

Members of the Tree of Life Core Informatics collective are listed here: https://doi.org/10.5281/zenodo.5013541.

Members of the Darwin Tree of Life Consortium are listed here: https://doi.org/10.5281/zenodo.4783558.
