# Supplementary material for: The genome sequence of the John Dory, Zeus faber Linnaeus, 1758
Source: Wellcome Open Res. 2024 Oct 22;9:150. Originally published 2024 Mar 19. [Version 2] doi: 10.12688/wellcomeopenres.21140.2 (PMC11179049; doi:10.12688/wellcomeopenres.21140.2)
Supplement: Peer review history

# Reviewer report: Reviewer response for version 2

**DOI:** 10.21956/wellcomeopenres.25627.r107167

**Published:** 2024-10-25

**Authors:** Naiara Rodriguez-Ezpeleta

**Affiliations:** 1 AZTI Basque Research and Technology Alliance, Bizkaia, Spain

The revised version is improved and ready for indexing.

Are sufficient details of methods and materials provided to allow replication by others?

Yes

Is the rationale for creating the dataset(s) clearly described?

Yes

Are the datasets clearly presented in a useable and accessible format?

Yes

Are the protocols appropriate and is the work technically sound?

Yes

Reviewer Expertise:

Population genomics, molecular evolution, high-throughput sequencing data analysis

I confirm that I have read this submission and believe that I have an appropriate level of expertise to confirm that it is of an acceptable scientific standard.

# Reviewer report: Reviewer response for version 1

**DOI:** 10.21956/wellcomeopenres.23384.r83999

**Published:** 2024-08-08

**Authors:** Nastaran Mazloumi

**Affiliations:** 1 University of Tasmania Institute for Marine and Antarctic Studies, Hobart, Tasmania, Australia

**Title:**

 The chosen title “The genome sequence of the John Dory, *Zeus faber* Linnaeus, 1758” lacks clarity and intention. You need to choose a better tile to define why you have done this study and what the genome sequestration is going to do!

**Background:**

 In the background section, this paragraph below is disjointed and very hard to follow:

 “John Dory is a commercially important species valued for human consumption, meal, and fish oil, as well as being important as a gamefish and in the aquarium trade (Iwamoto, 2015). It has been a prominent species in mixed species trawl fisheries in the British Isles and is a notable by-catch species for trawl gears globally (Dunn, 2001; Iwamoto, 2015). Despite being a highly regarded food fish, the only stock assessment for the British Isles, to our knowledge, was carried out between April 1994 and March 1996 (Dunn, 2001). Dunn describes landings from commercial fisheries in England and Wales alongside biological samples and catch data from the English Channel. John Dory was most abundant in the south and southwest of the British Isles (Dunn, 2001; Wheeler, 1969). Evidence suggests that the English Channel is a seasonal nursery ground for *Z. faber*: seasonal peaks in landings coincided with the period of recruitment, in this case during quarters three and four, when individuals were just over 1 year old at the transition between juvenile and adult life stages species (Dunn, 2001). Recruitment total length of the species was approximately 23 cm TL. Most commercial landings were in the range 23–29 cm TL with a maximum observed TL of 59 cm. Mean TL of first maturity was approximately 26 cm for males and 34.5 cm for females. The global conservation status of this species was last assessed in 2013, deemed ‘Data Deficient’ on the IUCN Red List (Iwamoto, 2015), which reflects the lack of historical data and biological information for this species (Dunn, 2001). Because of this, stock status and fishing pressure is uncertain for *Z. faber*”.

 This paragraph provides valuable information for reader but it significantly lacks clarity and confinement. For instance, human health issues with over the threshold elements in the flesh is one argument, data deficient stock assessment is another argument. Similarly, the genetically distinguished populations in different parts of the world that is discussed in the following paragraph, is another statement: “Molecular investigation of this species has shown significant genetic differentiation (7.44%) between clades in the North Atlantic/Mediterranean region and Australasia, indicating the possibility that they have speciated (Ward et al., 2008). The first genome of *Z. faber* was generated in 2016 for a study that suggests immune-related genes play an important role in teleost evolution and speciation (Malmstrøm et al., 2016). Here we present the second published genome of John Dory, collected and sequenced as part of the Darwin Tree of Life project (Blaxter et al., 2022). This dataset will be important for furthering our understanding of teleost pathology, immunology, evolution and phylogenetics (Malmstrøm et al., 2016; Ward et al., 2008)” – this confuse the reader as to why this study is important and why you made efforts to sequence genomes for John Dory.

 Background section, requires substantial revision in term of clarity and confinement and more importantly the message the author want to communicate with the reader as to why they have done this study and what gaps this is going to fill and how it serves the intention of the study. It needs to be specifically clarified as to whether this is for seafood industry and human health matter, or conservation matter? Or even management of the stock? What is the main intention here. without this valuable bit, it is not recommended to jump into the methodology and analysis.

**Summary:**

 Despite my encouragement for scientists to publish their work, I must admit that this particular research lacks clarity and conciseness. Therefore, I have no choice but to recommend rejection and suggest resubmission after thorough revisions, including expanding the research and writing in a more concise manner.

Are sufficient details of methods and materials provided to allow replication by others?

No

Is the rationale for creating the dataset(s) clearly described?

No

Are the datasets clearly presented in a useable and accessible format?

No

Are the protocols appropriate and is the work technically sound?

Partly

Reviewer Expertise:

Fisheries sciences, Biological Sciences, Climate Change, Fish Stock Assessment, influences of environmental and anthropogenic impacts on marine resources

I confirm that I have read this submission and believe that I have an appropriate level of expertise to state that I do not consider it to be of an acceptable scientific standard, for reasons outlined above.

# Response

**Published:** 2024-10-10

**Authors:** Tree of Life Team Sanger

**Affiliations:** Wellcome Trust Sanger Institute, UK

We respectfully disagree with the assertion that the title lacks clarity and intention. As this is a data note, the focus is specifically on presenting the genome sequence, which aligns with the journal’s scope for such submissions. Regarding the request for "thorough revisions, including expanding the research and writing in a more concise manner,” we believe there may be a misunderstanding of the purpose and structure of data notes. As stated in the journal's guidelines: “Data Notes are brief descriptions of scientific datasets that promote the potential reuse of research data and include details of why and how the data were created; they do not include any analyses or conclusions.” Expanding the research or adding detailed analysis would be beyond the scope of this format. We have, however, made edits to the introduction to enhance its clarity. Additionally, we note that the reviewer’s comments primarily address the introduction section and do not provide specific reasons for the assertion that the datasets are not presented effectively or that the work is technically unsound. We appreciate the feedback and have aimed to address the concerns raised within the boundaries of the data note format.

# Reviewer report: Reviewer response for version 1

**DOI:** 10.21956/wellcomeopenres.23384.r80568

**Published:** 2024-06-14

**Authors:** Naiara Rodriguez-Ezpeleta

**Affiliations:** 1 AZTI Basque Research and Technology Alliance, Bizkaia, Spain

This manuscript presents the genome sequence of a fish species (John Dory) that is in general poorly studied and for which conservation strategies can not be appropriately defined. Considering that understanding the genetic connectivity is critical for defining management units, and that full genomes facilitate the analysis and interpretation of population genomic data, this study is considered important for improving the conservation of the John Dory. The methods are well described and the results are pertinent. I have only one comment that I think the authors can address easily:

 - In the introduction they should better explain what is the knowledge of this species in terms of how many populations exist and potential subspecies. The data of  "significant genetic differentiation (7.44%) between clades in the North Atlantic/Mediterranean region and Australasia" seem to be taken from the abstract of a cited paper, but there is not much information here to understand what is the main issue that could be resolved with the help of a full genome sequence.

Are sufficient details of methods and materials provided to allow replication by others?

Yes

Is the rationale for creating the dataset(s) clearly described?

Yes

Are the datasets clearly presented in a useable and accessible format?

Yes

Are the protocols appropriate and is the work technically sound?

Yes

Reviewer Expertise:

Population genomics, molecular evolution, high-throughput sequencing data analysis

I confirm that I have read this submission and believe that I have an appropriate level of expertise to confirm that it is of an acceptable scientific standard.

# Reviewer report: Reviewer response for version 1

**DOI:** 10.21956/wellcomeopenres.23384.r79083

**Published:** 2024-05-10

**Authors:** Shawn M. Burgess

**Affiliations:** 1 National Human Genome Research Institute, Bethesda, USA

Adkins and colleagues have submitted a manuscript describing the genomic assembly of a John Dory fish ( *Zeus faber*). The sequencing was performed using PacBio HiFi and Hi-C data and the assembly using hifiasm and manual curation. The resulting assembly exceeds general benchmarks for high-quality assemblies including scoring for BUSCO, k-mer completeness, and percentage of sequence mapped to chromosomes. All raw and analyzed datasets are deposited in publicly available repositories. The data are clear, the approaches adequately documented. I have no substantial objections to any of the data presented. It should be a very useful assembly for researchers who are interested in John Dory biology.

Are sufficient details of methods and materials provided to allow replication by others?

Yes

Is the rationale for creating the dataset(s) clearly described?

Yes

Are the datasets clearly presented in a useable and accessible format?

Yes

Are the protocols appropriate and is the work technically sound?

Yes

Reviewer Expertise:

Zebrafish genetics and genomics. Fish genome assembly.

I confirm that I have read this submission and believe that I have an appropriate level of expertise to confirm that it is of an acceptable scientific standard.
